# Integration of G-Quadruplex and Pyrene as a Simple and Efficient Ratiometric Fluorescent Platform That Programmed by Contrary Logic Pair for Highly Sensitive and Selective Coralyne (COR) Detection

**DOI:** 10.3390/bios13040489

**Published:** 2023-04-19

**Authors:** Jiawen Han, Yaru Ding, Xujuan Lv, Yuwei Zhang, Daoqing Fan

**Affiliations:** 1 Laboratory for Marine Drugs and Bioproducts, National Laboratory for Marine Science and Technology, Key Laboratory of Marine Drugs, Ministry of Education, School of Medicine and Pharmacy, Ocean University of China, Qingdao 266003, China; 2 School of Pharmaceutical Sciences, Xiamen University, Xiamen 361102, China; 3 State Key Laboratory of Electroanalytical Chemistry, Changchun Institute of Applied Chemistry, Chinese Academy of Sciences, Changchun 130022, China

**Keywords:** ratiometric fluorescence, coralyne detection, contrary logic pair, G-quadruplex, anticancer drug

## Abstract

The effective and accurate detection of the anticancer drug coralyne (COR) is highly significant for drug quality control, medication safety and good health. Although various COR sensors have been reported in recent years, previous ones can only exhibit single-signal output (turn ON or turn OFF) with poor reliability and anti-interference ability. Therefore, exploring novel platform with dual-signal response for COR detection is urgently needed. Herein, we reported the first ratiometric fluorescent platform for highly sensitive and selective COR detection by integrating G-quadruplex (G4) and Pyrene (Py) as signal probes and harnessing A-COR-A interaction. In the absence of COR, the platform shows a low fluorescence signal of PPIX (F_642_) and a high one of Py monomer (F_383_). With the addition of COR, two delicately designed poly-A ssDNAs will hybridize with each other via A-COR-A coordination to form complete G4, yielding the increased fluorescence signal of PPIX and the decreased one of Py due to the formation of Py excimer. Based on the above mechanism, we constructed a simple and efficient sensor that could realize the ratiometric fluorescent detection of COR with high sensitivity and selectivity. A linear relationship between F_642_/F_383_ and COR’s concentration is obtained in the range from 1 nM to 8 μM. And the limit of detection of COR could reach to as low as 0.63 nM without any amplification, which is much lower than that of most COR sensors reported so far. Notably, the logical analysis of COR can be carried out under the control of a “YES-NOT” contrary logic pair, enabling the smart dual-channel response with an adequate S/N ratio and improved reliability and anti-interference ability. Moreover, this system also presents satisfactory performance in fetal bovine serum (FBS) samples.

## 1. Introduction

As a heterocyclic analogue of protoberberine alkaloids with a positive charge, coralyne (COR) has been proven to be an effective anticancer drug targeting human topoisomerase I with superior antitumor, antisecretory, anti-inflammatory and antimalarial functions [1,2,3,4,5]. However, its irrational use causes severe toxicity of lung, kidney, and other organs, along with the emergence of pathogen resistance [6,7]. Therefore, the effective and accurate detection of COR is of great significance for drug analysis, quality control, medication safety, etc. In recent decades, scientists have made continuous efforts to establish various methods for COR detection, such as high-performance liquid chromatography-electrospray ionization-mass spectrometry (HPLC-ESI-MS), fluorescence, colorimetry, electrochemical ones, etc. [8,9,10,11,12,13,14,15,16,17,18]. Although these strategies presented different advantages and disadvantages, to some extent, all of them pushed forward the development of COR sensors.

As a kind of oligonucleotide molecule with specific non-canonical structures or biological functions [19,20], functional DNA has become one of the most appealing biomolecules for biosensing because of its inherent merits of simple synthesis, easy modification, eminent programmability, etc. [21,22]. Among these, G-quadruplex (G4) is a unique secondary structure that derives from the stack of G-quartets (a kind of poly-G) [23,24], and it has been confirmed to possess some interesting properties. On the one hand, the fluorescence intensity of porphyrin dyes can be evidently enhanced by complete G4; on the other hand, the complex of G4/hemin (G4zyme) could exhibit high peroxidase-like activity [25,26]. Relying on the above principle, multifarious biosensors and bio-nanomachines for different targets can be subtly constructed. Furthermore, poly-T and poly-C DNAs have been used as ideal templates for the synthesis of metal nanoclusters [27,28]. Poly-A sequences are well-known substitutes for -SH during the linkage of AuNPs and DNA, and could also bind with COR via A-COR-A interaction to form an anti-parallel homo-adenine duplex [29,30]. Notably, based on the interaction between COR and poly-A sequences, researchers have developed different sensors to detect COR [31,32,33,34,35]. For example, Li et al. fabricated a label-free COR sensor by utilizing A-COR-A coordination and a split G4zyme, achieving the colorimetric detection of COR [36]. Ma et al. group realized the amplified fluorescent detection of COR based on a stimuli-responsive polymerase extension reaction [37]. Nevertheless, almost all the COR sensors reported until now can only present single-signal output with relatively poor reliability and anti-interference ability compared with dual-signal responsive sensors, and these drawbacks are likely to induce false positive/negative results. Therefore, it is necessary to explore a simple and universal system with a dual-signal output for COR detection.

The ratiometric fluorescence technique records the ratio of fluorescence values at two distinct wavelengths, and this strategy could perform cross-verification of the detection results via the concurrent positive and negative signals, which significantly improves the reliability while reducing potential background/environmental interferences [38,39]. Furthermore, molecular logic gates, with the unique advantages of accuracy, intelligence, and a binary-encoded digital output, have aroused scientists’ widespread interest and are widely applied to smart sensing, biomedical diagnosis, therapy, and other research fields [40,41,42,43,44]. For instance, Fan et al. proposed the concept of the DNA “contrary logic pair” (CLP, two parallel logic gates with opposite functions) and designed a universal system for operating various CLPs successfully [45]. It should be emphasized that the dual-channel mode of CLPs can not only bring a rigorous and intuitive digital output, but also significantly enhance reliability and accuracy, providing an elegant way to break the bottlenecks of current COR sensors [46,47,48,49]. However, as far as we know, ratiometric fluorescence has not been introduced into the analysis of COR, let alone the combination of COR detection with molecular logic computing. Against the above background, establishing a ratiometric fluorescent platform that controls by contrary logic pair (CLP) for highly sensitive and selective detection of COR is urgently needed.

Herein, by taking G4 as the universal building block, we for the first time introduced ratiometric fluorescence into COR’s detection, and constructed a simple and fast platform that programmed a “YES-NOT” contrary logic pair for the smart sensing of COR. Two delicately designed poly-A ssDNAs were modified with different parts of spilt-G4 (3:1 mode) at the 5′ and 3′ ends, whereas their 3′ and 5′ ends were labeled with two Pyrene units. In the absence of COR, the poly-A DNAs could not hybridize with each other, and the split-G4 parts did not enhance the fluorescence of PPIX, accompanied by the high fluorescence signal of the Py monomer, while, with the addition of COR, two ssDNAs would evolve into a duplex via A-COR-A coordination, and the formed complete G4 resulted in a high fluorescence intensity of PPIX. Meanwhile, the fluorescence of Py will be quenched because of the formation of a Py excimer, yielding a weak output signal of Py. Based on the above mechanism, a ratiometric fluorescent COR sensor was constructed reasonably. Moreover, this sensor presented high selectivity, and was applied to the analysis of COR in real samples.

## 2. Experimental Section

### 2.1. Chemicals

All the DNAs were synthesized by the Shanghai Sangon Biotechnology Co. Ltd. (Shanghai, China) and the corresponding sequences were listed in Table S1 (Supplementary Materials). Coralyne chloride, hemin, 3, 3′, 5, 5′-tetramethylbenzidine (TMB) and hydrogen peroxide (H_2_O_2_) were purchased from Sigma-Aldrich. Tris (hydroxymethyl) aminomethane (Tris) and potassium chloride (KCl) were provided by the Sinopharm Chemical Reagent Co., Ltd. (Shanghai, China). Protoporphyrin IX (PPIX), berberine, jatrorrhizine, 5-fluorouracil (5-FU), tobramycin (TOB), bleomycin (BLM) and memantine hydrochloride were purchased from Aladdin (Shanghai, China). 1 × Tris-HCl buffer (25 mM Tris, 50 mM KCl, pH 7.4) was used throughout the experiments and the distilled water was purified using a Millipore system.

### 2.2. Apparatus

The fluorescence spectra were measured on a F-4700 spectrofluorometer (Hitachi High-Tech Science Corporation, Tokyo, Japan). Corresponding parameters for the fluorescence spectra of PPIX and Py were as follows: Py (excitation wavelength: 340 nm, excitation and emission slit widths: 5 and 5 nm, respectively); PPIX (excitation wavelength: 410 nm, excitation and emission slit widths: 5 and 10 nm, respectively). Circular dichroism (CD) spectra were acquired on a JASCO J-815 CD spectropolarimeter. A solution of 1 × Tris-HCl buffer (25 mM Tris, 50 mM KCl, pH 7.4) was used to prepare the samples for CD tests. The spectra were collected in 1 mm path length cuvettes and the data were averaged from three parallel tests.

### 2.3. COR Detection and Selectivity Tests

For the detection of COR, all the DNA solutions (10 μM) were heated at 90 °C for 10 min and slowly cooled down to room temperature. Various concentrations of COR were mixed with 200 nM DNA in 1 × Tris-HCl buffer (25 mM Tris, 50 mM KCl, pH 7.4) and reacted for 10 min at room temperature. Different concentrations of PPIX were then added to the reaction system. Finally, a suitable volume of Tris-HCl buffer was added to make a total volume of 500 μL. After they had reacted in the dark for about 20 min, the fluorescence intensities were recorded immediately. For the selectivity experiments, 5 μM berberine, jatrorrhizine, 5-FU, TOB, BLM and memantine hydrochloride were used to replace the COR; all the other steps were similar to that of COR detection.

### 2.4. Recovery Tests of COR in Real Samples

To measure the recovery rate of the above COR sensor, fetal bovine serum (FBS) was diluted 100 times with 1 × Tris−HCl buffer (25 mM Tris, 50 mM KCl, pH 7.4) and taken as the biological medium of the COR. Different concentrations of COR in the linear range were spiked with above medium and added to the sensing system. The recovery rate was then tested according to the standard methods.

## 3. Results and Discussion

### 3.1. Sensing Mechanism of the Ratiometric Fluorescent System

Before illustrating the mechanism of the ratiometric fluorescent system in this work, we would like to elaborate our initial design principle. As we mentioned above, dual-signal responsive sensors for COR have been barely reported, though various single-signal ones have been constructed so far [50]. The main speculated reason can be attributed to the intrinsic fluorescence emission of COR at 475 nm, which can be further enhanced after binding with poly-A sequences. The emission spectra of COR may overlap with those of many frequently used fluorescent dyes, making it very difficult to find another two non-interfering fluorescent probes at the same time. To resolve this problem, we speculate that exploring one probe whose emission wavelength is much shorter than 475 nm and that of another probe whose emission wavelength is much longer than 475 nm may be a potential strategy. Moreover, to achieve the ratiometric changes, the two probes should exhibit inverse responses in the presence of COR. 

**Figure 1:**
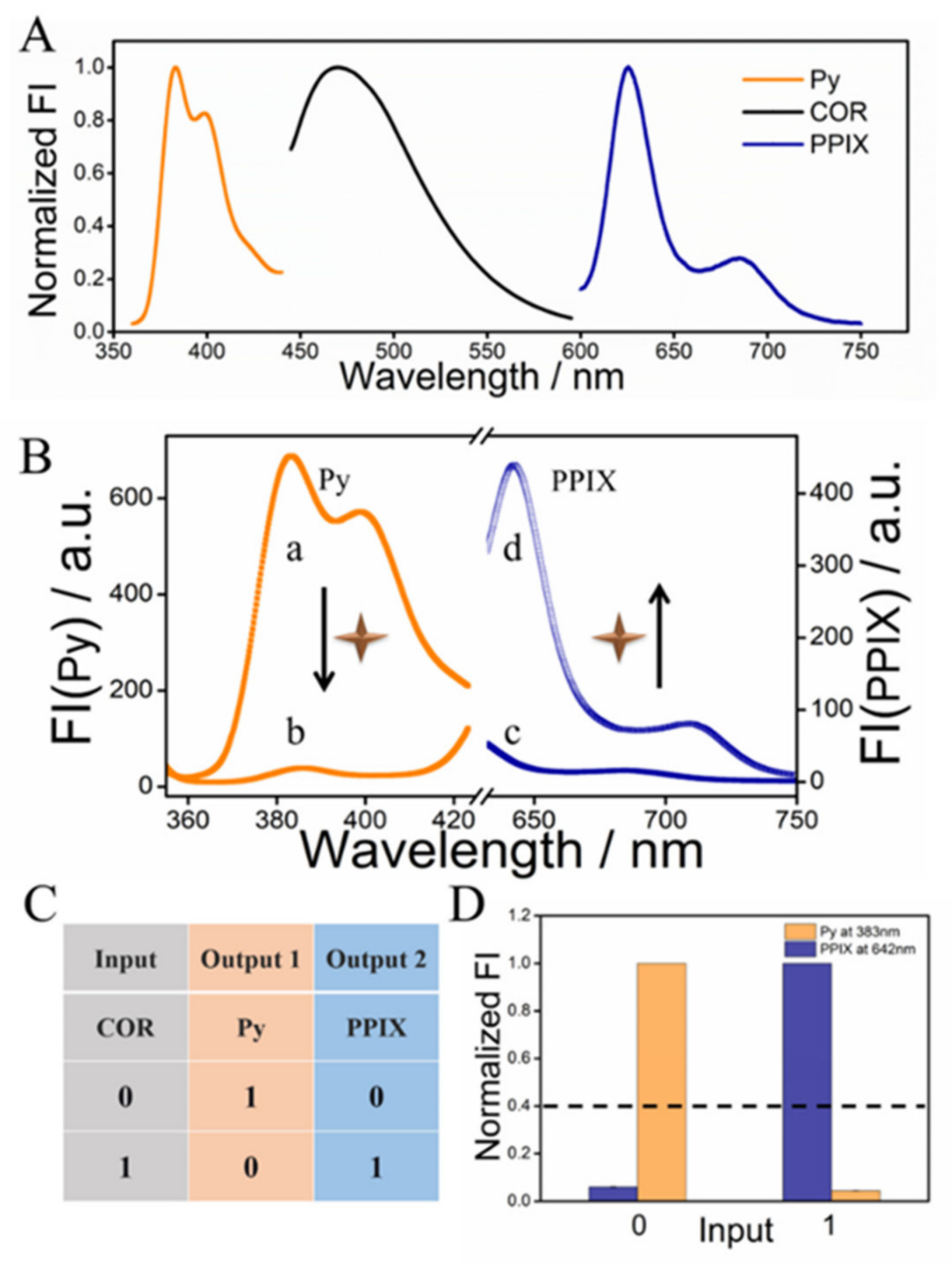
(**A**) The emission spectra of Py, COR and PPIX; (**B**) Fluorescence spectra of Py and PPIX in the absence and presence of COR, curve a/c: G1-A10-Py (200 nM) + Py-A10-G3 (200 nM), curve b/d: G1-A10-Py (200 nM) + Py-A10-G3 (200 nM) + COR (5 μM); (**C**) Truth table of “YES-NOT” contrary logic pair; (**D**) Normalized Py and PPIX column bars of the YES-NOT pair under different input states; “0” represents the absence of COR and “1” represents the presence of COR. The error bars are obtained via three independent experiments.

**Scheme 1:**
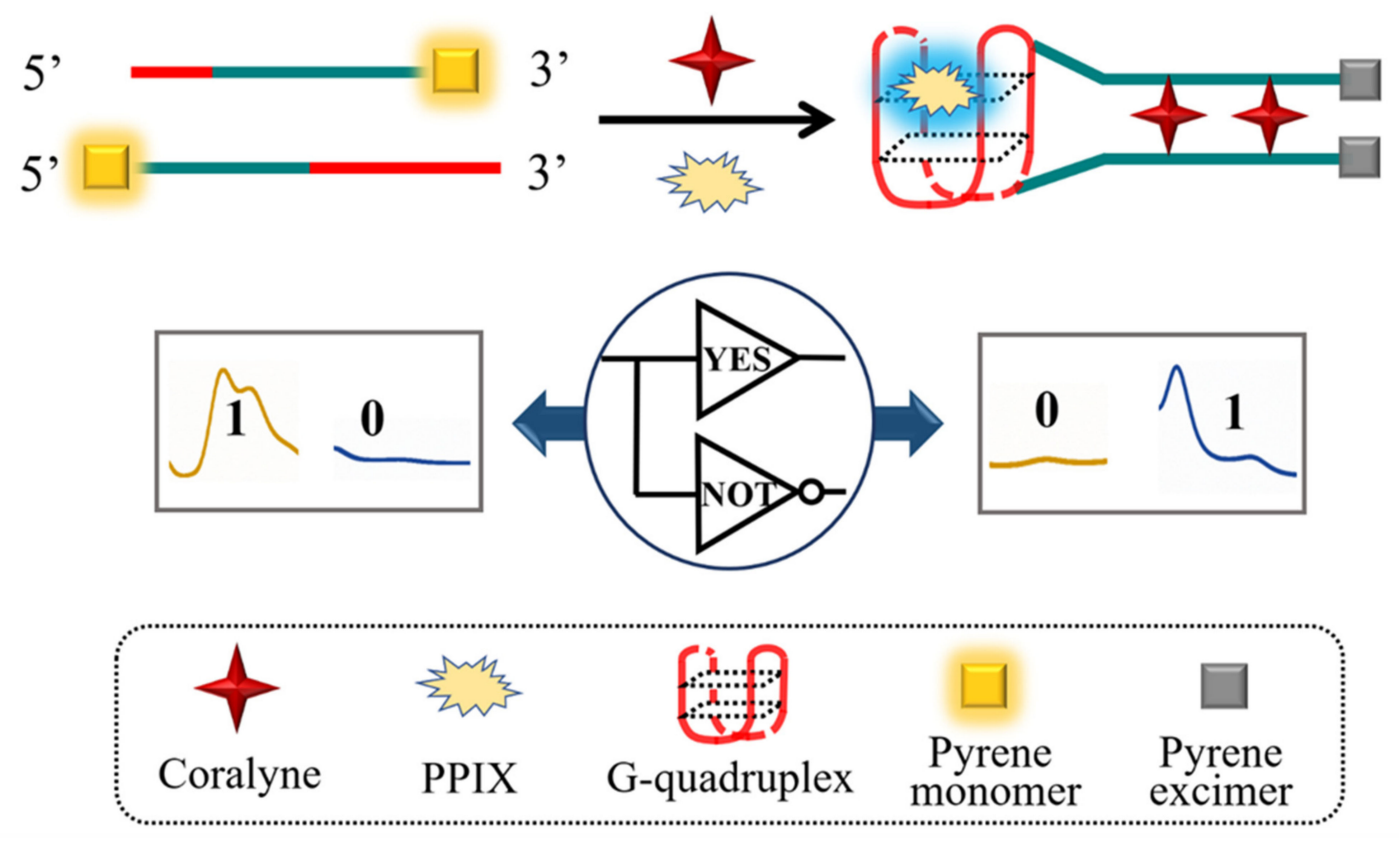
Mechanism of the ratiometric fluorescent platform that programmed by contrary logic pair for highly sensitive and selective detection of COR.

Keeping the above principle in mind, we chose pyrene (Py, emission peak at around 383 nm for monomer) and PPIX (emission peak at 642 nm) as potential ratiometric fluorescent probes according to our previous scientific experience and attempted to design a simple and fast system for ratiometric COR sensing. Figure 1A presents the emission spectra of Py, COR and PPIX; there were no obvious interferences or overlaps between each one of them, proving the potential feasibility of our above design principle. Scheme 1 illustrates the sensing mechanism of the ratiometric fluorescent sensor. To elaborate it clearly, two delicately designed poly-A (green parts) ssDNAs were modified with different parts of the spilt-G4 (3:1 mode) at the 5′ and 3′ ends (red parts), whereas their 3′ and 5′ ends were labeled with Py groups, respectively. As a spatially sensitive fluorescent dye, Py monomer could yield high fluorescence at around 383 nm with two characteristic peaks. However, two ssDNAs would hybridize with each other via A-COR-A coordination if COR was introduced, resulting in the formation of Py excimer that has an emission peak at 485 nm (which we do not use as an output signal because of its overlap with COR’s emission spectra) [51,52,53]. With the addition of COR, the concentration of Py monomer decreased, leading to a decrease in its fluorescence at 383 nm. Accordingly, the fluorescence signal of PPIX will be evidently enhanced by the formed G4. The ratiometric fluorescent changes in the absence/presence of COR were then realized. All the above illustrated the sensing mechanism of this sensor, and the ratiometric fluorescent detection of COR can be achieved by recording the fluorescence changes of PPIX and Py monomer (F_642_/F_383_).

### 3.2. Verification of the Proposed Sensing Mechanism

**Figure 2:**
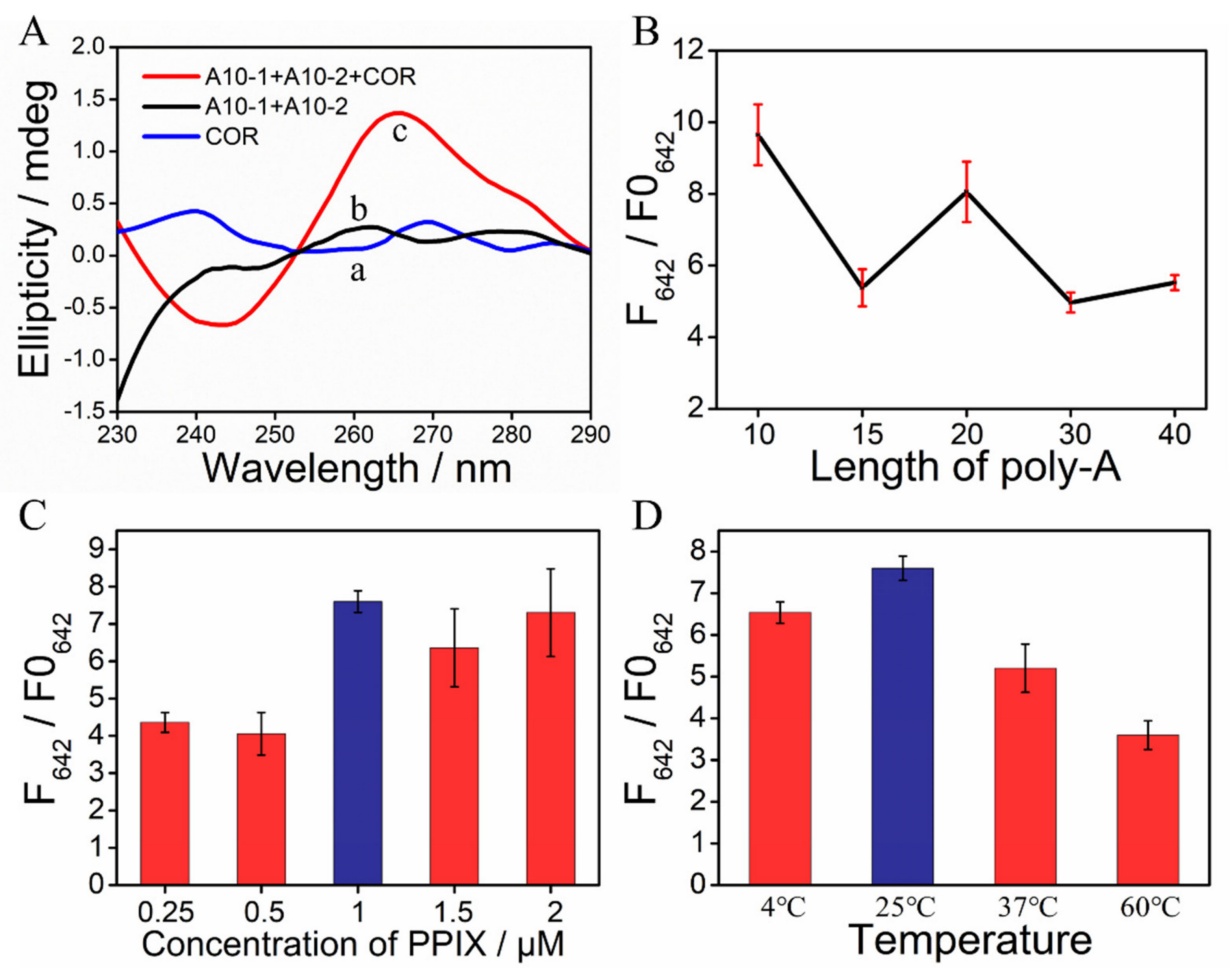
(**A**) CD spectra of (a) solely COR and A10 strands in the (b) absence and (c) presence of COR; (**B**) Optimization of the length of poly−A; (**C**) Optimization of the concentration of PPIX; (**D**) Optimization of the reaction temperature. The error bars are obtained via three independent experiments.

As shown in Figure 1B, the feasibility of this platform was further validated by fluorescent experiments. A weak fluorescence signal of PPIX at 642 nm and a strong one of Py monomer at 383 nm were generated when only two ssDNAs existed in the reaction system. However, the addition of 5 μM COR caused the formation of an A-COR-A duplex and the subsequent generation of Py excimer, greatly decreasing the concentration of Py monomer and consequently yielding a weak fluorescence intensity of Py monomer at 383 nm. At the same time, the formed G4 will evidently enhance the fluorescence signal of PPIX at 642 nm. The ratiometric changes of two signal probes in the absence/presence of COR were vividly achieved. To prove the valid A-COR-A interaction, a pair of widely used Forster resonance energy transfer (FRET) fluorophores, FAM and BHQ1, was labeled at the 5′ and 3′ ends of two poly-A ssDNAs. In the absence of COR, a high fluorescence signal of FAM can be observed (Figure S1A). Meanwhile, after adding COR into the system, FAM’s fluorescence was dramatically quenched because of the well-known FRET effect between FAM and BHQ1 (Figure S1B) [54]. The corresponding interaction of two poly-A ssDNAs can be clearly identified by the new band that appeared during the native-PAGE analysis (Lane 4, Figure S2). Moreover, the formation of G4 was further confirmed by corresponding CD spectra, as illustrated in Figure 2A. In the presence of solely COR, no peaks can be observed, Figure 2A(a). In the absence of COR, the two ssDNAs could not induce any obvious peaks in the CD spectra, Figure 2A(b). Meanwhile, after the addition of COR, a positive peak at 265 nm and a negative peak at 243 nm were observed, Figure 2A(c) indicates the formation of parallel G4 [55]. Furthermore, the peroxidase-like property of G4 DNAzyme was also used to confirm the formation of G4 (See Figure S3). The distinct yellower color of TMB and obvious absorbance enhancements after the addition of enough COR further demonstrated the formation of G4 via A-COR-A interaction. All these results fully proved the feasibility of our proposed strategy.

Furthermore, a molecular logic operation was integrated with the ratiometric fluorescent detection of COR for the first time. It should be noted that the detection was achieved under the control of a “YES-NOT” contrary logic pair, enabling the smart logical analysis of COR via the dual-channel response with high reliability and accuracy. As shown in Scheme 1, Figure 1C,D, two ssDNAs were taken as the platform of the logic device. COR was used as the input (absence “0,” presence “1”), and the fluorescence signal of Py monomer and PPIX were two contrary outputs (low “0,” high “1”). In the absence of COR, the high fluorescence signal of Py (F_383_) and low one of PPIX (F_642_) corresponded to the output “1-0”. Meanwhile, in the presence of COR, the resulting low signal of Py (F_383_) and high one of PPIX (F_642_) corresponded to the output “0-1”. This phenomenon featured the characteristics of the “YES-NOT” contrary logic pair properly, which can be further identified by the fluorescent column bars in Figure 1D, in which the threshold value was set at 0.40 to distinguish the positive/negative outputs. According to the truth table (Figure 1C), the existence state of COR can be easily identified, endowing the following COR detection with the accuracy and reliability of dual-output Boolean logic. 

### 3.3. Optimization of Reaction Conditions

In order to achieve the best experimental conditions, various factors that may affect the sensing performance were optimized, including the length of poly-A, the concentration of PPIX, the temperature and pH. 

Firstly, the length of the homo-adenine chain was studied. Because the detection of COR is based on the interaction between COR and poly-A, the length of poly-A is crucial. By taking the fluorescence intensity of PPIX as a signal, we testified the effects of the poly-A length. As displayed in Figure 2B, the number of poly-A varied from 10 to 40 nucleotides, in which the strands can be denoted as A10, A15, A20, A30, and A40. The results showed that all DNAs with split-G4 parts could enhance the fluorescence of PPIX in the presence of COR, and A10 generated the best signal-to-noise (S/N) ratio (corresponding fluorescence spectra of PPIX are shown in Figure S4 (SI), Supporting Information (SI)). Hence, A10 was used as the optimal strand in subsequent experiments.

The concentration of PPIX was further optimized by using split-G4 modified A10 strands. Additionally, the fluorescence intensities of PPIX in the presence and absence of COR were measured. As depicted in Figure 2C, we could obtain the satisfactory S/N ratios when the concentration of PPIX was 1 μM. Thus, 1 μM PPIX was selected in the following detection, and the corresponding fluorescence spectra of PPIX with/without COR after adding different contents of PPIX are given in Figure S5 (SI), respectively. Finally, the temperature and pH were also optimized in a similar way. Taking the S/N ratio and universality into account, the temperature of 25 °C and Ph = 7 were chosen as the optimum conditions, as depicted in Figure 2D and Figure S6 (SI). In addition, the detection of COR in this platform was operated under the above circumstances.

### 3.4. Ratiometric Fluorescent Detection of COR

**Figure 3:**
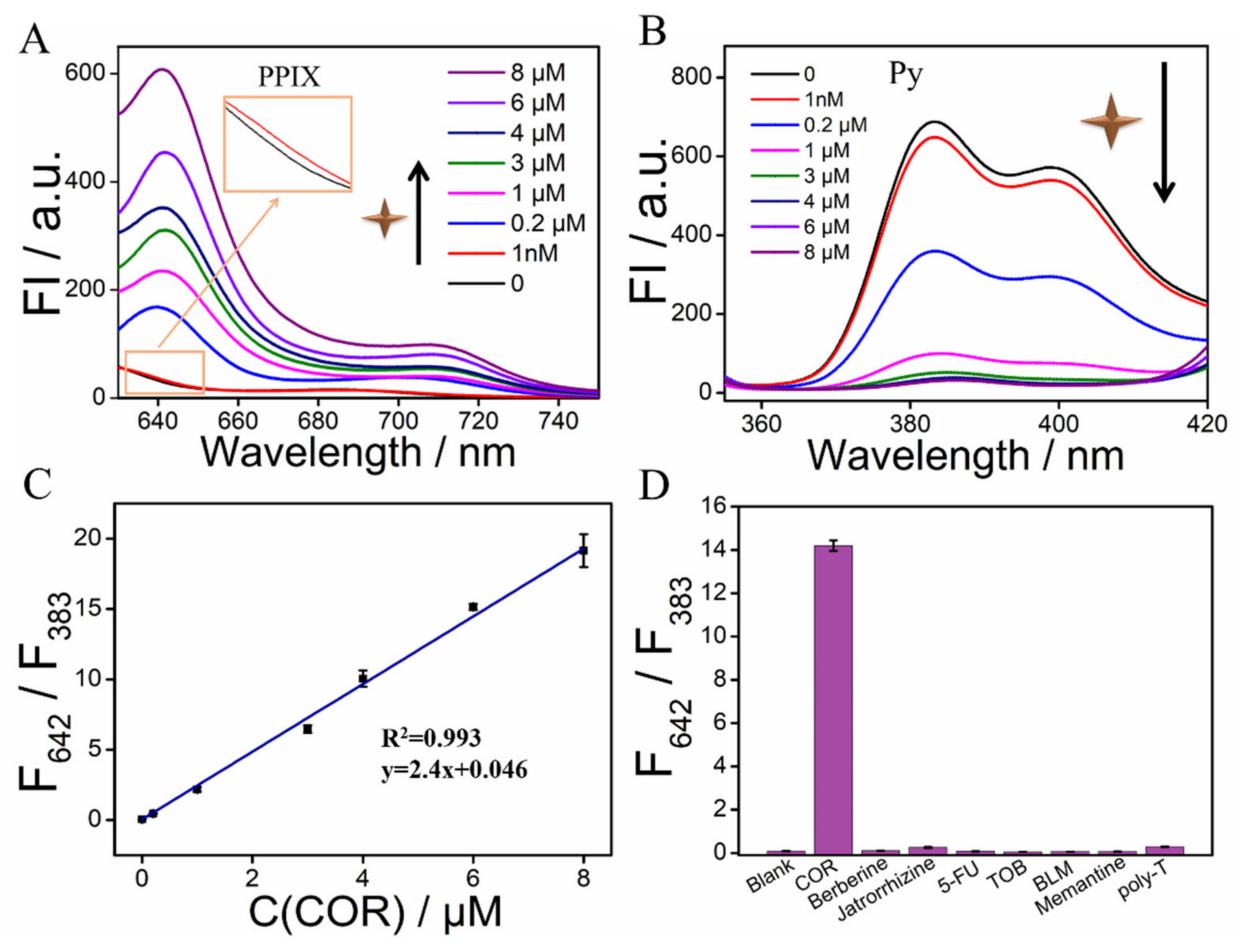
(**A**) Fluorescence spectra of PPIX in the presence of different concentrations of COR; (**B**) Fluorescence spectra of Py in the presence of different concentrations of COR; (**C**) Linear relationship between the fluorescence ratio F_642_/F_383_ and the concentration of COR; (**D**) Comparison of the fluorescence ratio F_642_/F_383_ with other alkaloids, drugs and poly-T strands for this system. The error bars are obtained via three independent experiments.

Under the optimal experimental conditions, the ratiometric fluorescent detection of COR was then implemented by recording the fluorescence intensity of PPIX and Py upon the addition of varying contents of COR. As displayed in Figure 3A,B, the fluorescence signal of PPIX increased gradually with the elevated concentration of COR, while that of Py monomer decreased (the complete fluorescence spectra of Py are shown in Figure S7, SI). As depicted in Figure 3C, the F_642_/F_383_ ratio values were proportional to the concentration of COR. In addition, a linear relationship between F_642_/F_383_ and COR’s contents was obtained in the range from 1 nM to 8 μM. The limit of detection (LOD) of COR was calculated to be 0.63 nM through the 3σ rule, which is much lower than most previous COR sensors (Table 1). It should be noted that there are no amplification techniques in this system, indicating the high performance of the fabricated sensor. The error bars further indicated the excellent reproducibility of the detection results. Thus, the ratiometric fluorescent detection of COR based on the above mechanism was properly accomplished. 

In addition, some researchers may suspect that the reason why we do not use the unique ratiometric changes of solely Py’s two kinds of emission (F_485_ for excimer and F_383_ for monomer) as the opposite outputs and conduct the ratiometric COR detection. The detailed explanations can be elaborated as follows: (1) the emission spectra of Py excimer will overlap with those of inherent COR, bringing unexplained interferences, especially after mixing COR with poly-A strands; (2) the relevant calibration curves between F_485_/F_383_ or F_383_/F_485_ as a function of various concentrations of COR were also measured (Figure S8 in SI), and we did not obtain any ideal linear relationships that could compete with that of the above constructed sensor. All above phenomena fully indicated the rationality and ingenuity of our design.

### 3.5. Selectivity and Real Sample Application of the System

After the sensitive detection of COR, the selectivity of our biosensor was further examined, in which the interaction between COR and poly-A acted as the key factor. To evaluate the selectivity of the above ratiometric fluorescent sensor, the influences of other alkaloids (berberine and jatrorrhizine), 5-fluorouracil (5-FU, an anticancer agent), tobramycin (TOB), bleomycin (BLM, antibiotic drugs) and memantine hydrochloride (a drug for treating senile dementia) on the fluorescence of PPIX and Py were evaluated. As presented in Figure 3D, under the same test conditions, 5 μM COR led to the highest F_642_/F_383_ values, whereas other chemicals with the same concentration can only bring negligible ratiometric values because of their weaker interaction with poly-A strands. Moreover, if poly-A strands were replaced with poly-T strands, no obvious ratiometric changes in PPIX and Py were observed (Figure 3D and Figure S9). All the above phenomena indicated the excellent selectivity of our system for COR detection.

**Figure 4:**
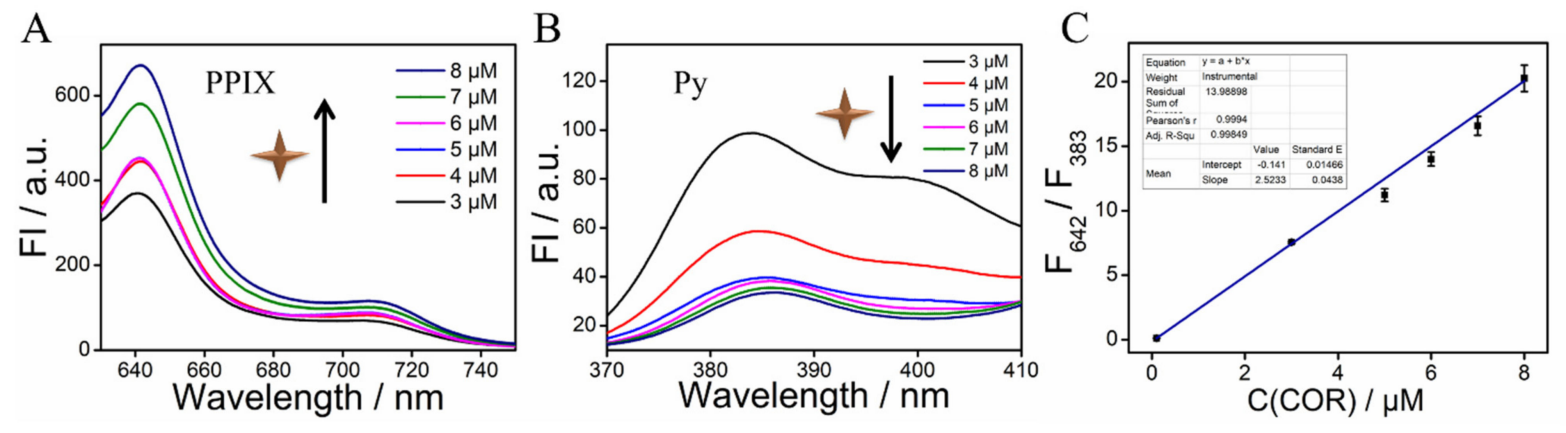
(**A**) Fluorescence spectra of PPIX in the presence of various concentrations of COR by using 1% FBS as the reaction solution; (**B**) Fluorescence spectra of Py in the presence of various concentrations of COR by using 1% FBS as the reaction solution; (**C**) Linear relationship between the F_642_/F_383_ ratio and COR’s concentrations in the range from 100 nM to 8 μM by using 1% FBS as the reaction solution. The error bars are obtained via three independent experiments.

Finally, the practicability of the designed system for COR detection in real samples was investigated. The recovery tests were performed by spiking different contents of COR with 1% FBS (diluted 100 times with Tris-HCl buffer). As depicted in Figure 4A,B, the fluorescence signal of PPIX increased gradually with the addition of spiked COR, while that of Py decreased. In addition, a good linear relationship between the F_642_/F_383_ ratio and CORs concentrations was achieved in the range from 100 nM to 8 μM by using 1% FBS as the reaction solution (Figure 4C). (Complete fluorescence spectra of Py are shown in Figure S10, SI.) Corresponding results are summarized in Table 2; the obtained recovery rates ranged from 99.8% to 106.7% for the ratiometric sensor, suggesting a satisfactory performance of the above system in real sample applications.

## 4. Conclusions

In summary, we for the first time introduced ratiometric fluorescence and molecular logic computing into COR detection, and successfully constructed a simple and efficient platform for operating a COR sensor based on G4 and Pyrene. In contrast to previous works, this work presented several advantages. Firstly, the detection was achieved under the control of a “YES-NOT” contrary logic pair, enabling smart logical analysis of COR with improved reliability and accuracy. Secondly, the LOD of COR reached as low as 0.63 nM for the above sensor without any amplification, which was much lower than that of most fluorescent COR sensors reported until now. Finally, this ratiometric fluorescent platform exhibited excellent selectivity and showed satisfactory performance in spiked FBS samples. This study not only broke the bottlenecks of current COR biosensors and significantly improved the reliability and accuracy while reducing the background interferences, but also provided inspiring prototypes for the design of other anticancer drug sensors.

## Figures and Tables

**Table 1:** Comparison of different methods for the detection of COR.

Detection Methods	LOD	Liner Range	Ref.
Colorimetric	91 nM	0–728 nM	[14]
Colorimetric	100 nM	0.1–1 μM	[15]
Colorimetric	19 nM	0.06–10 μM	[16]
Fluorescent	10 nM	10–700 nM	[9]
Fluorescent	0.31 nM	0.2–100 nM	[10]
Fluorescent	5.8 nM	0.01–5 μM	[11]
Fluorescent	3.1 nM	1–300 nM	[12]
Fluorescent	3.5 nM	10–1000 nM	[13]
Fluorescent	0.98 nM	2–500 nM	[37]
Electrochemical	0.07 nM	0.1–100 nM	[17]
SERS	100 nM	0.1–100 μM	[18]
Ratiometric fluorescent	0.63 nM	0.001–8 μM	This work

**Table 2:** Recovery test results of this sensor in fetal bovine serum (FBS) samples.

Sample	Added	Measured	Recovery (%)	RSD (%)
COR	100 nM	99.8 ± 2.88 nM	99.8	2.9
	4 μM	4.27 ± 0.24 μM	106.7	5.5
	8 μM	8.02 ± 0.37 μM	100.3	4.6

## Data Availability

All the data generated in this study is included in this article itself.

## Supplementary Materials

The following supporting information can be downloaded at: https://www.mdpi.com/article/10.3390/bios13040489/s1, Table S1: DNA sequences used in this work; Figures S1, S4, S5, S7 and S10: Fluorescence spectra of PPIX, FAM and Py during the experiments; Figures S2 and S3: Evidence of the formation of G4 via A-COR-A interaction; Figures S6 and S8: Optimization and validation experiments; Figure S9: Poly-T interference.

## Abbreviations

COR	Coralyne
G4	G-quadruplex
FRET	Fluorescence resonance energy transfer
LOD	Limit of detection
S/N ratio	Signal-to-noise ratio
ss-DNA	Single-stranded DNA
Tris	Tris (hydroxymethyl) aminomethane
5-FU	5-fluorouracil
BLM	Bleomycin
HPLC-ESI-MS	High-performance liquid chromatography-electrospray ionization-mass spectrometry
TMB	3, 3′, 5, 5′-Tetramethylbenzidine
Py	Pyrene
G4zyme	G-quadruplex DNAzyme
PPIX	Protoporphyrin IX
AuNPs	Gold nanoparticles
CLP	Contrary logic pair
ds-DNA	Double-stranded DNA
KCl	Potassium chloride
TOB	Tobramycin
CD	Circular dichroism
FBS	Fetal bovine serum
H_2_O_2_	Hydrogen peroxide
