# Supplementary material for: Integration of G-Quadruplex and Pyrene as a Simple and Efficient Ratiometric Fluorescent Platform That Programmed by Contrary Logic Pair for Highly Sensitive and Selective Coralyne (COR) Detection
Source: Biosensors (Basel). 2023 Apr 19;13(4):489. doi: 10.3390/bios13040489 (PMC10136222; doi:10.3390/bios13040489)
Supplement: Supplementary file 1 [file biosensors-13-00489-s001.zip › biosensors-2263166-supplementary.pdf]

Supporting Information

Integration of G-Quadruplex and Pyrene as a Simple and Efficient Ratiometric Fluorescent Platform that Programmed by Contrary Logic Pair for Highly Sensitive and Selective Coralyne (COR) Detection

Jiawen Han [1], Yaru Ding [2], Xujuan Lv [1], Yuwei Zhang [1] and Daoqing Fan [1,3,*]

[1] Laboratory for Marine Drugs and Bioproducts, National Laboratory for Marine Science and Technology, Key Laboratory of Marine Drugs, Ministry of Education, School of Medicine and Pharmacy, Ocean University of China, Qingdao 266003, China

[2] School of Pharmaceutical Sciences, Xiamen University, Xiamen 361102, China

[3] State Key Laboratory of Electroanalytical Chemistry, Changchun Institute of Applied Chemistry, Chinese Academy of Sciences, Changchun 130022, China

* Correspondence: fdq9688@ouc.edu.cn or dqfan93@163.com

TABLE OF CONTENT

Experiment section.....3

Table S1.....4

Figure S1.....5

Figure S2.....6

Figure S3.....7

Figure S4.....8

Figure S5.....9

Figure S6.....10

Figure S7.....11

Figure S8.....12

Figure S9.....13

Figure S10.....14

Experiment Section

Colorimetric experiments

Colorimetric experiments of TMB catalyzed by A-COR-A G4zyme were performed as follows: 1 × HEPES buffer (25 mM HEPES, 10 mM NaCl, 0.05 % (w/v) TritonX-100, 1% (v/v) DMSO, pH 7.4) and 1 × MES-HAc buffer (25 mM MES, 10 mM KAc, pH 4.5) were used in colorimetric experiments. 500 nM DNA and the suitable concentration of COR were incubated in 1 × HEPES buffer for 30 min, then, 500 nM hemin was added. The above mixture was reacted at room temperature for 1 h to form A-COR-A G4zyme. After that, 20 μL G4zyme solution was used in TMB-H_2O_2 reaction system. 5 μL TMB (0.5 % (w/v)), 5 μL H_2O_2 (30 %) and 1 × MES-HAc buffer were successively added at last to make a total volume of 500 μL. After 5 min, 5 μL H_2SO_4 was added to terminating the reaction. Finally, UV-vis absorption spectra were recorded on a Shimadzu UV-2600i spectrophotometer.

Table S1. DNA sequences used in the work.

Strand	Sequence (5’ to 3’)
Py-A10-G3	Py-AAAAAAAAAAGGGTAGGGCGGG
G1-A10-Py	TGGGTAAAAAAAAAA-Py
G1-A10-FAM	TGGGTAAAAAAAAAA-6-FAM
BHQ1-A10-G3	BHQ1-AAAAAAAAAAGGGTAGGGCGGG
G1-A10	TGGGTAAAAAAAAAA
A10-G3	AAAAAAAAAAGGGTAGGGCGGG
G1-A15	TGGGTAAAAAAAAAAAAAAA
A15-G3	AAAAAAAAAAAAAAAGGGTAGGGCGGG
G1-A20	TGGGTAAAAAAAAAAAAAAAAAAAA
A20-G3	AAAAAAAAAAAAAAAAAAAAGGGTAGGGCGGG
G1-A30	TGGGTAAAAAAAAAAAAAAAAAAAAAAAAAAAAAA
A30-G3	AAAAAAAAAAAAAAAAAAAAAAAAAAAAAAGGGTAGGGCGGG
G1-A40	TGGGTAAAAAAAAAAAAAAAAAAAAAAAAAAAAAAAAAAAAAAAA
A40-G3	AAAAAAAAAAAAAAAAAAAAAAAAAAAAAAAAAAAAAAAAGGGTAGGGCGGG
G1-T10	TGGGTTTTTTTTTTT-Py
T10-G3	Py-TTTTTTTTTTGGGTAGGGCGGG

Figure S1

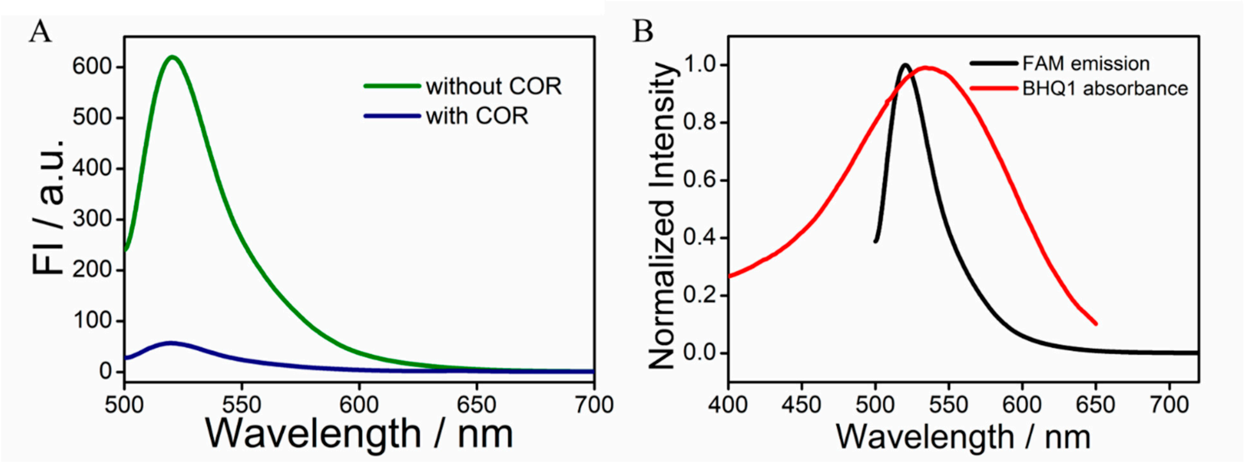

Figure S1. (A) Fluorescence spectra of FAM in the absence and presence of COR; (B) UV-vis spectra of BHQ1 (red line) and fluorescence spectra (black line) of FAM.

Figure S2

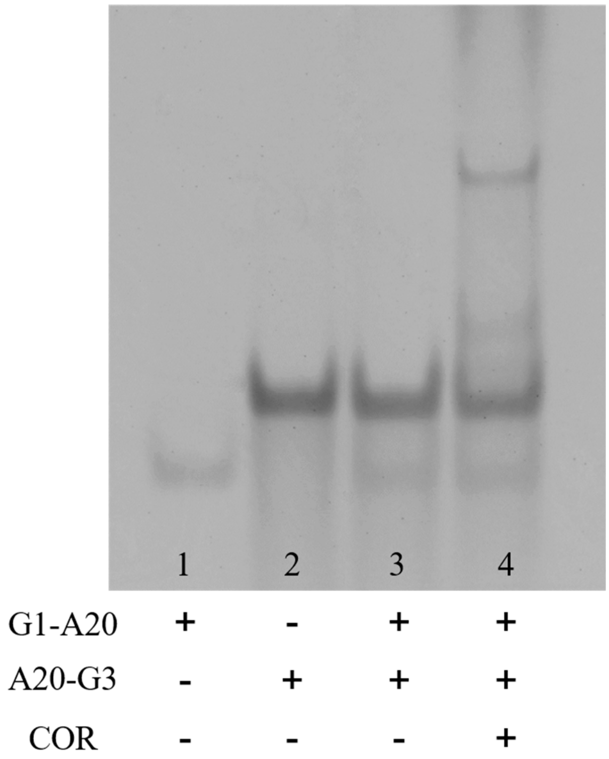

Figure S2. PAGE characterization of A-COR-A interaction. Lane 1 to 4 corresponds to G1-A20, A20-G3, G1-A20+A20-G3, G1-A20+A20-G3+COR, respectively. The concentrations of G1-A20, A20-G3 and COR are 20 μM, 5 μM, 10 μM, respectively. The electrophoresis experiments were conducted in 15% native polyacrylamide gel at the voltage of 80 V.

Figure S3

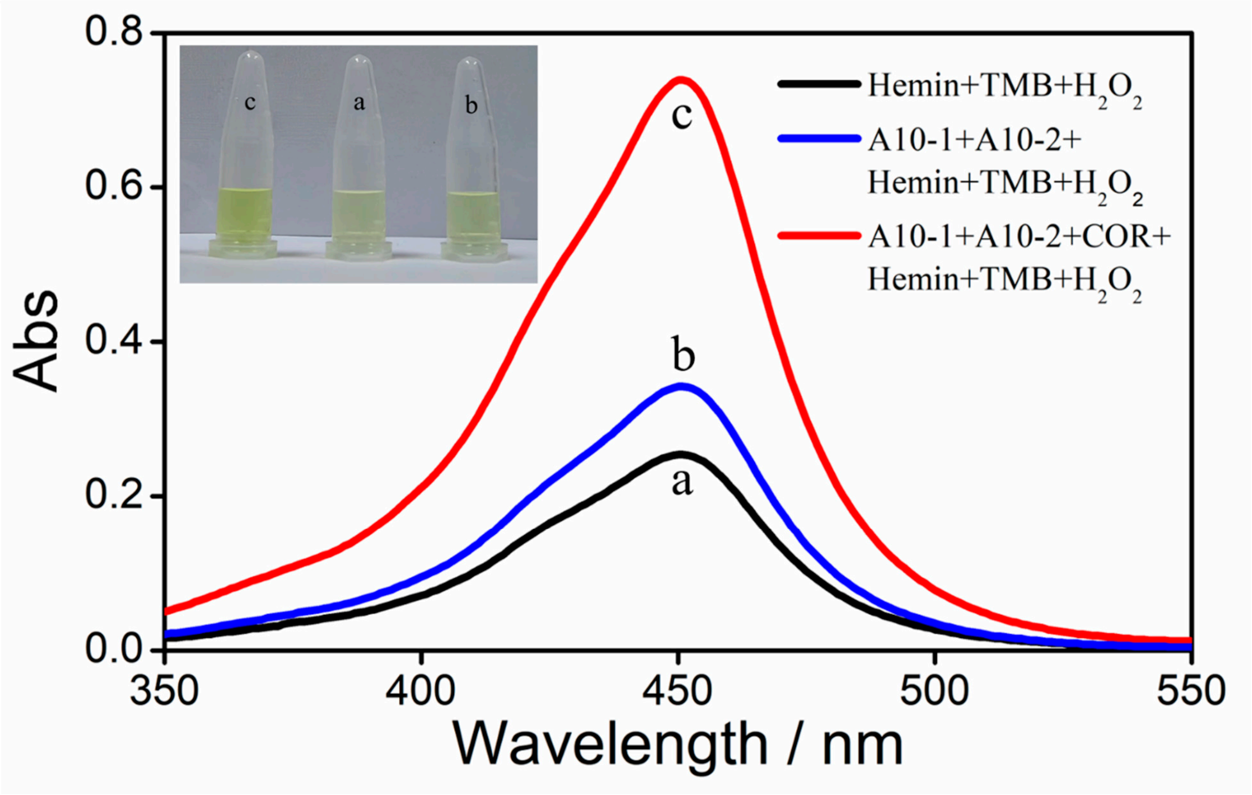

Figure S3. UV-vis absorption spectra of the TMB-H_2O_2 reaction system. (a) Hemin+TMB+H_2O_2; (b) A10-1+A10-2+Hemin+TMB+H_2O_2; (c) A10-1+A10-2+COR+Hemin+TMB+H_2O_2.

Figure S4

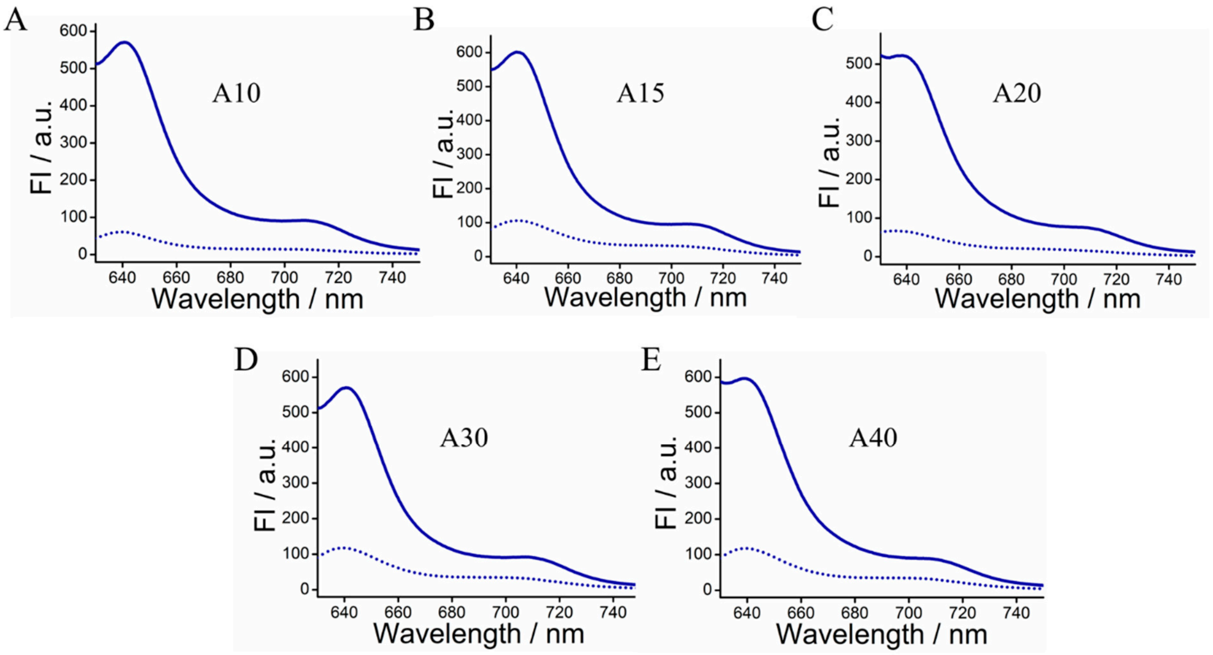

Figure S4. Fluorescence spectra of PPIX corresponding to the optimization experiment of poly-A's length in the absence (dotted lines) and presence (solid lines) of COR. (A) A10; (B) A15; (C) A20; (D) A30; (E) A40. The concentrations of PPIX and COR in this experiment are 1.5 μM and 5 μM, respectively.

Figure S5

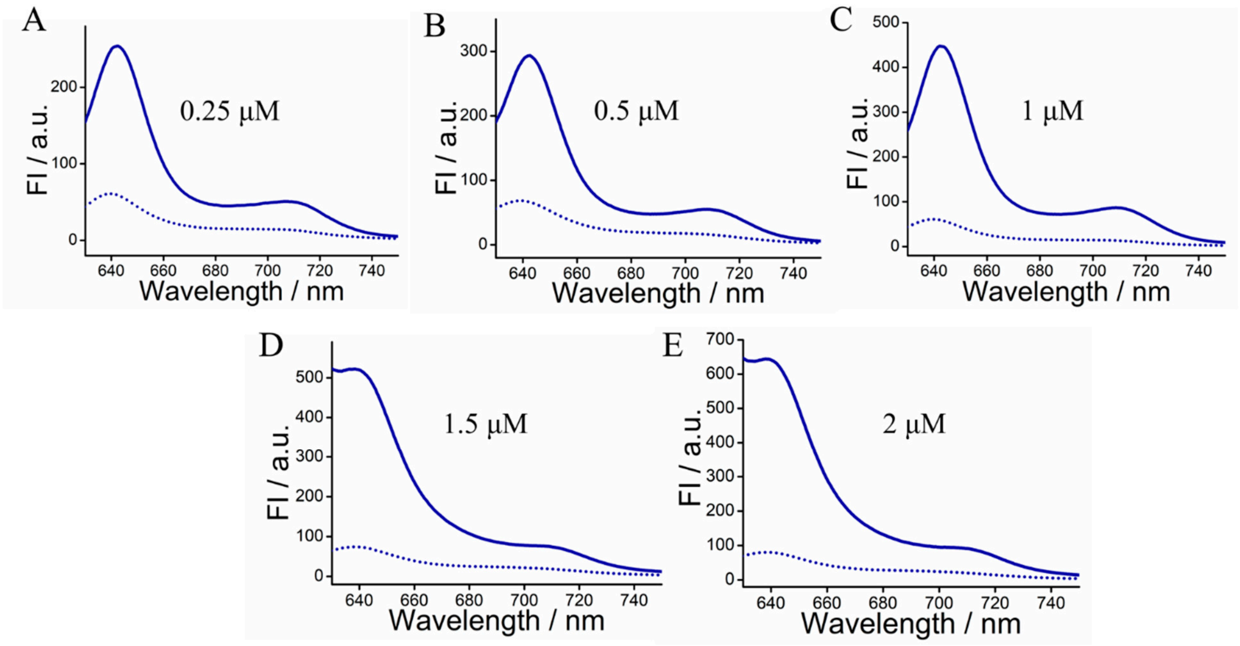

Figure S5. Fluorescence spectra of PPIX corresponding to the optimization experiment of PPIX's concentration in the absence (dotted lines) and presence (solid lines) of COR. (A) 0.25 μM; (B) 0.5 μM; (C) 1 μM; (D) 1.5 μM; (E) 2 μM. The concentration of COR in this experiment is 5 μM and the poly-A strands are two A10s, respectively.

Figure S6

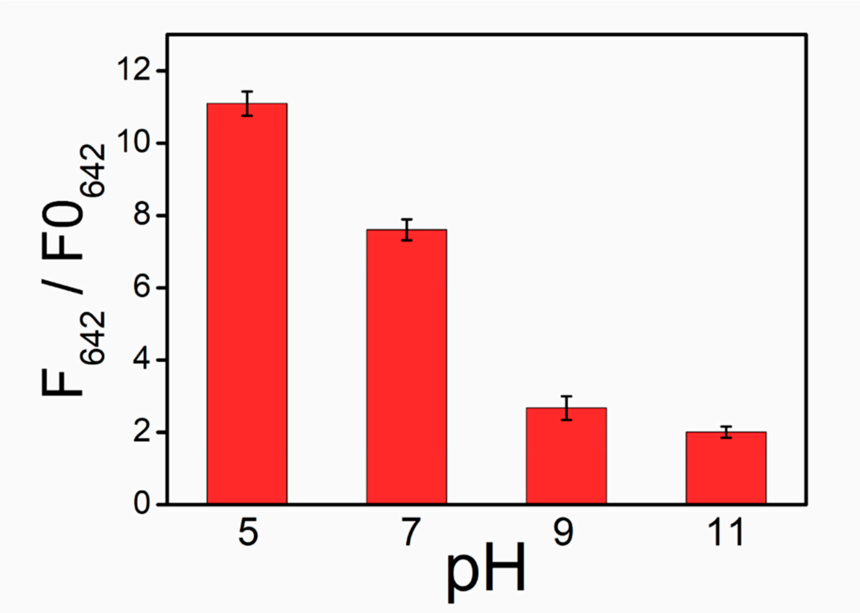

Figure S6. Optimization of the pH. The concentrations of PPIX and COR in this experiment are 1 μM and 5 μM, respectively. The poly-A strands are two A10s, respectively. The error bars are obtained via three independent experiments.

Figure S7

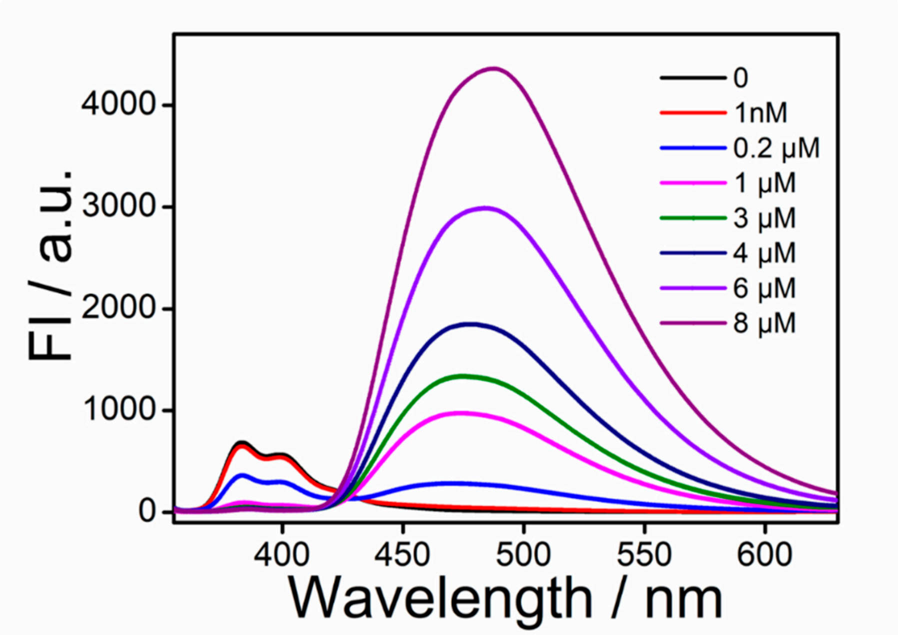

Figure S7. The complete fluorescence spectra of Py with increasing concentrations of COR.

Figure S8

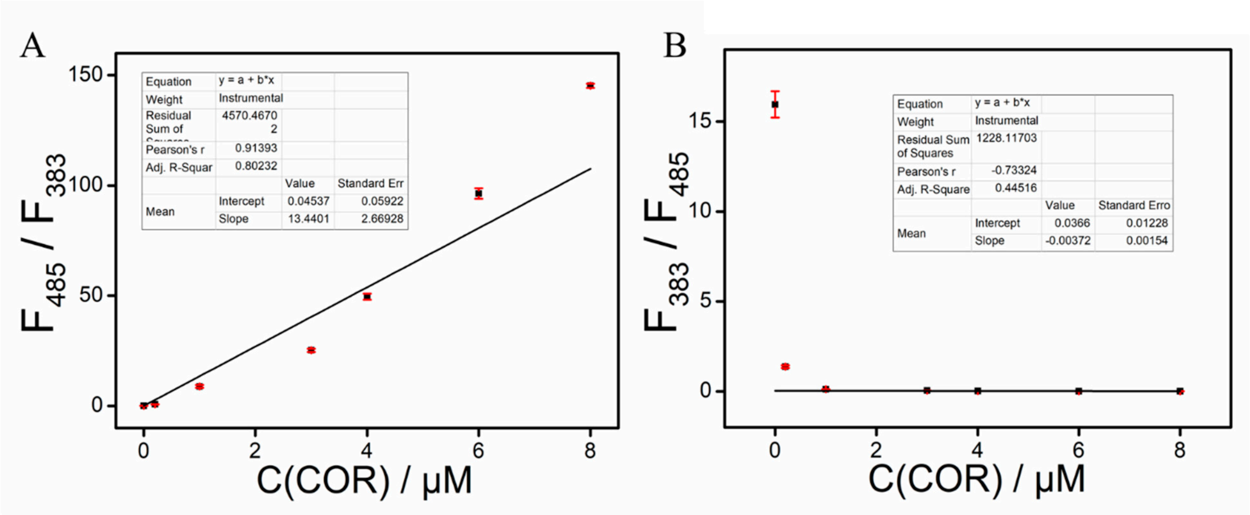

Figure S8. The relevant calibration curves between (A) F_{485}/F_{383} and (B) F_{383}/F_{485} as a function of various concentrations of COR. The error bars are obtained via three independent experiments.

Figure S9

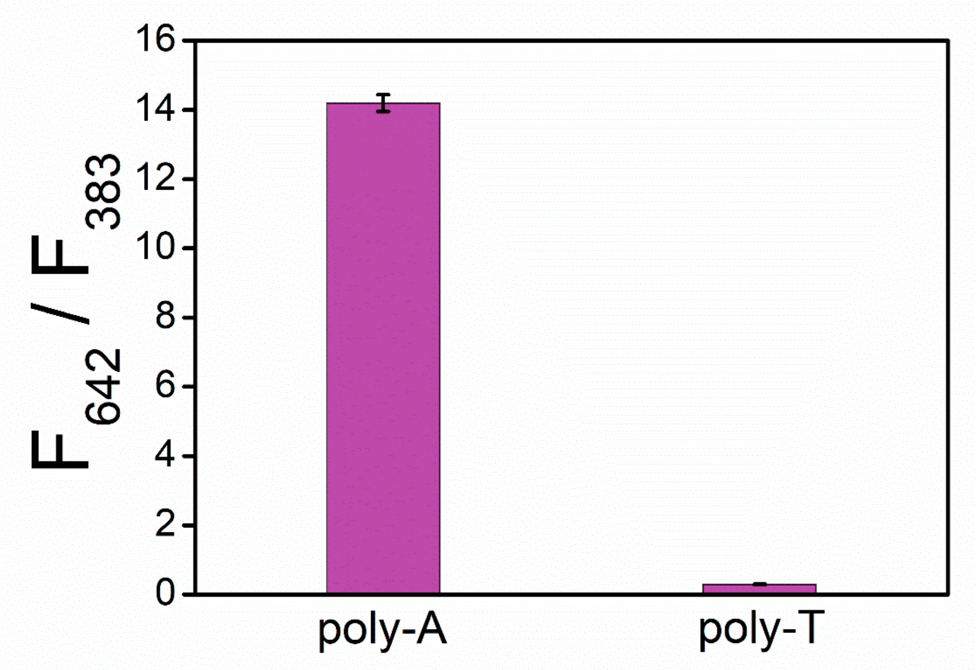

Figure S9. Ratiometric fluorescent columns with error bars in the presence of poly-A and poly-T for this system.

Figure S10

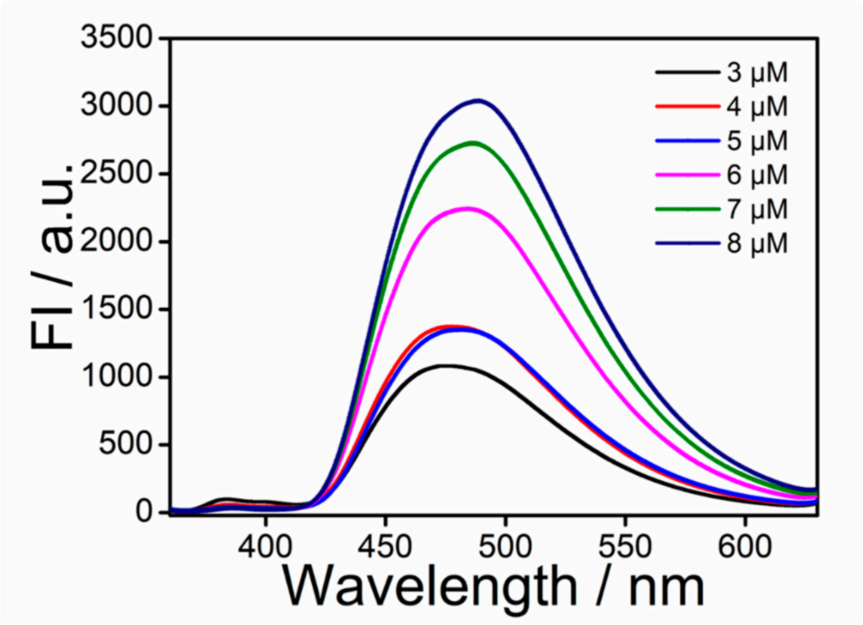

Figure S10. The complete fluorescence spectra of Py in real sample application.
